# Dr. Byron Hiram Daggett

**Published:** 1904-02

**Authors:** 


### OBITUARY.

Dr. Byron Hiram Daggett, of Buffalo, died at the General Hospital, December 30, 1903, after a gallstone operation, aged 62 years. Dr. Daggett was born at Girard, Pa., received his preliminary education at Allegheny College, attended a medical course at the University of Michigan, and took his doctorate degree from the medical department of the University of Buffalo in 1867. He began the practice of medicine immediately in Buffalo, and continued to be so engaged until his death. He served two terms as health physician, beginning in 1869, and 8 years as police surgeon, beginning in 1876. He was also for a number of years surgeon of the 74th Regiment, N. G. N. Y.; was a member of the staff of the Emergency Hospital and of the Buffalo Hospital of the Sisters of Charity; and also was attending surgeon at the Erie County Hospital. Of late years he turned his attention chiefly to genitourinary medicine and surgery and achieved much success in the practice of this specialty. It was in this branch that he served in the hospitals above named.

Among his other accomplishments Dr. Daggett was a man of mechanical ingenuity, having invented many instruments and appliances, valuable not only in his own special branch but useful also in other fields of medicine or surgery. The Daggett examining table in use among surgeons and gynecologists is one of the best yet devised, and serves as an example of Dr. Daggett's ingenious turn of mind.

Dr. Daggett was a companionable man, full of wit, biting sarcasm and sparkling anecdote, one or all of which he could use with effect when occasion required. The various phases of his character have been masterfully dealt with in a memorial which we publish elsewhere in this issue of the JOURNAL. For some years he conducted the department of genitourinary surgery in the JOURNAL, and occasionally original contributions were offered from his facile pen. Dr. Daggett is survived by a widow and two sons.

